# Relationship between the double burden of malnutrition and mental health in overweight and obese adult women

**DOI:** 10.1017/jns.2022.7

**Published:** 2022-02-21

**Authors:** Mohammad Gholizadeh, Leila Setayesh, Habib Yarizadeh, Atieh Mirzababaei, Cain C. T. Clark, Khadijeh Mirzaei

**Affiliations:** 1 Department of Cellular and Molecular Nutrition, School of Nutritional Sciences and Dietetics, Tehran University of Medical Sciences, Tehran, Iran; 2 Department of Community Nutrition, School of Nutritional Sciences and Dietetics, Tehran University of Medical Sciences (TUMS), Tehran, Iran; 3 Centre for Intelligent Healthcare, Coventry University, Coventry, CV1 5FB, U.K

**Keywords:** Anxiety, Depression, Double burden of malnutrition, Stress, BMI, body mass index, DBM, double burden of malnutrition, DBP, vitamin D binding protein, 25(OH)D, 25-hydroxy vitamin D

## Abstract

The co-existence of overweight or obesity with concurrent deficiency of one or more nutrients is referred to as double burden of malnutrition (DBM), and numerous mental health impairments have been associated with a variety of nutrient deficiencies. Although DBM is relevant for several health outcomes, the ubiquitous involvement of vitamin D across multiple systems and tissues suggests D insufficiency as a viable target for nutritional modification. The present study aimed to evaluate the contribution of DBM and mental health among adult women. Study participants included 300 women, aged 18–59 years, who presented to one of the 25 health centres in Tehran. Participants with a body mass index (BMI) of greater than 25 kg/m^2^ and a plasma concentration of 25-hydroxy vitamin D [25(OH)D] of >20 ng/ml were considered to have DBM. The 147-item food frequency questionnaire was used to estimate their dietary intake. Mental health status was assessed using the depression, anxiety and stress scales-21 (DASS-21). The mean ± standard deviation age, weight and BMI of the participants were 36⋅49 ± 8⋅38, 80⋅89 ± 12⋅45 kg and 31⋅04 ± 4⋅31 kg/m^2^, respectively. DBM was significantly associated with stress, after adjusting for potential confounders, including age, energy and marital status in model 1 (OR = 1⋅28, 95 % confidence interval (CI) 1⋅00, 1⋅65, *P* < 0⋅04) *v.* the crude model (OR = 1⋅22; 95 % CI 0⋅96, 1⋅55, *P* = 0⋅09). No significant association was seen among DBM and DASS-21 outcomes. In this cross-sectional study, stress and DBM were significantly associated. While vitamin D insufficiency was associated with mental health and obesity in opposing directions. Elucidation of whether vitamin D supplementation can improve mental health impairments requires further evaluation.

## Introduction

The continued rise in overweight and obesity remains a global health concern. In particular, the increases and consequent adverse health effects widely recognised among developing countries are introducing a significant health challenge^(1)^. Furthermore, the emergence of double burden of malnutrition (DBM), the co-existence of caloric overconsumption with essential nutrient insufficiency, has been reported as increasingly prevalent in developing countries in comparison to developed countries^(2,3)^. While contributors to this phenomenon include increased urbanisation, alteration of dietary patterns and lifestyle^(4)^, some nutrients are more likely to be implicated in adverse health effects.

The physiologic effects of DBM are well recognised. However, a comprehensive understanding of modifiable health outcomes requires an understanding of the psychological effects of DBM. Indeed, an association between obesity and a number of mental health impairments across the life course has been reported^(5,6)^.

Given that poor mental health affects more than 300 million people, of all ages, the additive effect of the obesity epidemic and mental health impairments could have a profound effect on mortality in the ensuing decade(s). Indeed, a bidirectional link between obesity and mental health has been suggested^(7)^, with obesity increasing the risk of depression incidence, and a suggested mechanistic role^(8)^ of inadequate essential nutrient intake as a moderator^(9,10)^. Suboptimal nutrient intake, including cobalamin, folic acid, niacin and ascorbic acid, zinc, iron and selenium, has been associated with depression^(11)^. However, the ubiquitous involvement of vitamin D in neurocognition and obesity-related disorders offers a viable area of investigation as a modifiable intervention^(12,13)^. Many studies have reported that changes in a plasma concentration of 25-hydroxy vitamin D [25(OH)D] and fatty mass were inversely and significantly associated; moreover, obese subjects tended to show low absorption of vitamin D^(14,15)^. Furthermore, vitamin D absorbs by complex mechanisms; for instance, in the gastrointestinal tract (GIT), vitamin D is involved in lipid metabolism and diffuses in chylomicrons, which facilitates transportation in the liver^(16)^. Also, obese subjects demonstrate a low concentration of plasma 25(OH)D, as well as low 25(OH)D and obesity related to high waist circumference, high body mass index (BMI), hypertension, insulin resistance, glycaemic profile, impaired lipids and other cardiometabolic disorders^(17,18)^. Several explanations have been offered to explain the reduced concentration of 25(OH)D, including low sun exposure because of a sedentary lifestyle, the sequestration of 25(OH)D in adipose tissue, and dilution of ingested 25(OH)D and synthesis in large fat of obese patients^(19)^. Furthermore, BsmI and ApaI VDR genes in overweight and obese women are reported to account for polymorphism and seem to affect BMI. Also, adipose stores have a haemostatic role in inflammation and innate immunity in human individuals by modulating of toll-like receptors and nuclear factor-kappaB (NF-ĸB) pathways^(20–22)^.

Observational studies have suggested that vitamin D deficiency may be related to depression and increased suicide^(23–26)^. However, there is a distinct dearth of studies about the association between DBM and mental health. Therefore, in this cross-sectional study, we assessed the association between DBM and mental health, including depression, anxiety and stress, in Iranian women with obesity, with a focus on vitamin D as a potential moderator.

## Method

### Study design

A cohort of 300 women with obesity and overweight, aged 18–59 years, participated in the present study. Women were randomly selected from those presenting to twenty-five health centres in Tehran. The sampling method was achieved by multistage cluster randomisation among all the Tehran regions. The participants were included among twenty clusters from 2017 to 2019.

Inclusion criteria were aged 18–59 years; no current weight loss programme; no use of weight loss supplements. Participants with a history of type 2 diabetes, cardiovascular diseases, polycystic ovary syndromes, stroke, non-alcoholic fatty liver disease, inflammatory disease, hypertension, cancer, thyroid diseases, or who were currently pregnant, were excluded because of possible changes in diet. In addition, women reporting intake energy lower or higher than 800–4200 kcal, respectively, were excluded.

The present study was conducted according to the guidelines in the Declaration of Helsinki and all procedures involving human subjects were approved by the ethics committee of the Tehran University of Medical Science (IR.TUMS.VCR.REC.1398.819). Written informed consent was obtained from all subjects prior to participation.

### Anthropometric assessments

The individuals wore light clothing and were unshod for the measurement of weight and height. All measurements were assessed by a trained technician. The waist circumference^(27)^ was measured using a non-elastic tape, to the nearest 0⋅1 cm, according to standard protocols. In addition, hip circumference was determined as the largest part of the hip, over light clothing. BMI was calculated based on Quetelet's index^(28)^. According to WHO guidelines, the individuals were classified as underweight (BMI: <18⋅50 kg/m^2^), normal weight (18⋅50–24⋅99 kg/m^2^), overweight (25⋅0–29⋅9 kg/m^2^) and obesity (≥30⋅0 kg/m^2^).

#### Complete body composition analysis

The body composition of all participants was analysed using a body analyser device (model BC-418, MA-Tanita, product by the UK). This instrument measured the body composition, including body fat mass, fat-free mass (FFM)^(29)^ and visceral fat area (VFA), using bioelectrical impedance analysis^(30)^. According to the manufacturer's instructions, after shoes, coats and sweaters had been removed, subjects were required to stand on the balance scale, in bare feet, and hold the handles of the machine. The measurements took approximately 20 s, and the output was printed.

### Dietary intake assessment

The individual diet intake was assessed by a 147-item food frequency questionnaire that was previously validated^(31)^. The data documented in household measures and serving size were changed to grams and millimetres. The nutritionist IV (First Data Bank, San Bruno, CA) food analyser was used for analysing dietary intake.

### Blood sampling and biochemical parameters

At first, a 10-ml blood sample was drawn after 8–12 h of fasting, and the serum was centrifuged, and isolated and stored at −80°C. All data were evaluated by the Endocrinology & Metabolism Research Institute (EMRI) Bionanotechnology laboratory of the Tehran University of Medical Science. Serum fasting glucose assessment was made using glucose oxidase–phenol 4-amino antipyrine peroxidase (GOD-PAP) (colorimetric method), while triacylglycerol (TG) and total cholesterol^(20)^ (CHOL) were measured using glycerol-3-phosphate oxidase–phenol 4-amino antipyrine peroxidase (GPO-PAP) and enzymatic endpoint, respectively. Also, low-density lipoprotein (LDL) and high-density lipoprotein (HDL) cholesterol were measured by direct enzymatic clearance assay. All laboratory assessments were performed using Seven Randox Laboratories kits (Random Laboratories Ltd., Ardmore, UK).

The concentration of 25(OH)D in individuals was assessed using an immunoassay immunodiagnostic system (IDS) kit. Finally, serum concentrations of 25(OH)D between 20 and 30 ng/ml and less than 20 ng/ml were considered as insufficiency and deficiency, respectively^(32–35)^.

#### Calculation of the DBM and mental disorders

DBM was assessed by calculating BMI (based on Quetelet's index^(28)^) and vitamin D levels. Accordingly, BMI greater than 25 kg/m^2^ along with plasma concentrations of vitamin D lower than 20 ng/ml (25(OH)D < 20 ng/ml) was considered DBM.

#### Depression, anxiety and stress scale questionnaire

Mental health status was calculated using the depression, anxiety and stress scales^(36)^, which is a 42-item self-report scale that generates three scale scores, with each scale consisting of 14 questions scored from 0 to 3. Where 0 = did not apply to me at all and 3 = applied to me very much, or most of the time, with a total possible scale range for each scale of 0–42^(37,38)^.

### Statistical analysis

Normality distribution was tested using a Kolmogorov–Smirnov test; for data that were not normally distributed, and could not be transformed appropriately for normal distribution, *z*-scores were used. Data on quantitative characteristics were reported as the mean ± sd, and data on qualitative characteristics were expressed as a percentage. χ^2^ analysis was performed for detection confounders and to recognise mental health disorders. One-way analysis of variance (ANOVA) and Tukey *post hoc* tests, where appropriate, were used for a comparison of variables. The logistic regression was performed to discern the relationship between vitamin D deficiency in overweight and people with obesity by mental disorders. An initial model (0) was created and included only vitamin D deficiency (using 20 as a cut point), and a secondary model (1) included vitamin D deficiency and mental health status, in addition to age, energy and marital status. All statistical analysis was performed using IBM SPSS for Windows version 23 (SPSS Inc, Chicago, IL, USA), and a *P* value of <0⋅05 was, *a priori*, considered as statistically significant.

## Results

Descriptive statistics are represented in Table 1. Accordingly, the mean age and BMI of participants were 36⋅49 ± 8⋅38 years and 31⋅04 ± 4⋅3 kg/m^2^, respectively.

**Table 1.** Baseline characteristic of participants (mean ± sd)

Name (n) Mean		SD
**Age^a^**	**36.47**	**8.40**
Overweight^b^ (*n* 137)	35.21	7.97
Obese (*n* 161)	37.54	8.64
**Weight (total)**	**80.81**	**12.39**
Overweight (*n* 138)	72.15	6.37
Obese (*n* 161)	88.24	11.44
**BMI (kg/m^2^)**	**31.04**	**4.31**
Overweight (*n* 139)	27.57	1.51
Obese (*n* 162)	34.02	3.67

Body composition		
**BFM**	**34.04**	**8.69**
Overweight (*n* 139)	27.42	3.66
Obese (*n* 162)	39.72	7.68
**WHR**	**1.23**	**5.24**
Overweight (*n* 139)	0.90	0.04
Obese (*n* 162)	1.51	7.15
**WC**	**99.01**	**10.05**
Overweight (*n* 139)	91.48	5.53
Obese (*n* 162)	105.48	8.42
**Visceral fat area (cm^2^)**	168.30	103.11
Overweight (*n* 139)	133.87	23.94
Obese (*n* 162)	197.84	131.97

Blood parameters and blood pressure		
**CHOL (g/dl)**	**185.29**	**35.85**
Overweight (*n* 126)	177.47	31.63
Obese (*n* 128)	192.98	38.14
**TG (g/dl)**	**122.52**	**69.38**
Overweight (*n* 126)	104.07	47.63
Obese (*n* 128)	140.67	81.77
**HDL (mg/dl**)	**46.63**	**10.80**
Overweight (*n* 126)	47.55	10.15
Obese (*n* 128)	45.72	11.38
**LDL (mg/dl)**	**95.53**	**24.07**
Overweight (*n* 126)	92.80	22.44
Obese (*n* 128)	98.21	25.38
**SBP (mm Hg)**	**111.75**	**13.69**
Overweight (*n* 133)	108.88	12.66
Obese (*n* 152)	114.25	14.10
**DBP (mm Hg)**	**77.88**	**9.60**
Overweight (*n* 133)	75.86	8.59
Obese (*n* 152)	79.65	10.15
**25(OH)D (ng/ml)**	**66.74**	**47.00**
Overweight (*n* 109)	69.23	46.74
Obese (*n* 124)	64.54	47.32

^a^ table items indicate the variables in overweight and obese in totals as well as ^b^ in overweight and obese participants separately. BMI, body mass index; BFM, body fat mass; FFM, fat-free mass, WHR, waist hip ratio; WC, waist circumference; TG, triacylglycerol; HDL, high-density lipoprotein; LDL, low-density lipoprotein; SBP, systolic blood pressure; DBP, diastolic blood pressure.

The number of people in each category and characteristics are shown in Table 2. Based on Table 2, 305 individuals were initially enrolled in the present study; however, after exclusion based on our inclusion/exclusion criteria, 231 individuals remained for analysis. In Table 2, we show that there were no significant differences in variables between groups.

**Table 2.** Association between mental health and sample sizes in each confounder

Variables		Anxiety (*n* 282)			Stress (*n* 282)			Depression (*n* 282)		
		cat1 (*n*)	cat2 (*n*)	*P*	cat1 (*n*)	cat2 (*n*)	*P*	cat1 (*n*)	cat2 (*n*)	*P*
Education	Illiterate	1	2	0⋅57	1	2	0⋅34	1	2	0⋅48
	Primary education	3	10		5	8		3	10	
	Intermediate Education	6	12		3	13		5	13	
	High school education	5	3		6	1		5	3	
	Diploma	33	51		44	40		44	40	
	Postgraduate education	13	13		12	14		13	13	
	Bachelor's degree and higher	52	78		65	65		78	52	
Job	Housekeeper	70	101	0⋅51	86	85	0⋅39	83	88	0⋅19
	Labour	2	2		2	2		2	2	
	Management employee	21	25		8	28		25	21	
	Non-managerial employee	12	22		22	12		25	9	
	Household jobs	4	5		8	9		4	5	
	University student	5	13		8	12		10	8	
Marriage	Married	89	130	0⋅29	106	113	0⋅04	113	106	0⋅14
	Single	23	31		30	24		34	20	
	Away from spouse more than 6 month	0	2		0	2		0	2	
	Dead spouse	1	1		2	0		1	2	
	Divorce	0	5		0	5		0	4	
Monthly salary	Low	5	5	0⋅26	2	4	0⋅69	2	4	0⋅49
	Medium	4	8		7	6		6	7	
	Good	36	76		43	74		42	71	
	Excellent	53	95		61	87		68	82	
Housing status	Owner	74	97	0⋅17	92	79	0⋅11	92	79	0⋅64
	Tenant	38	63		43	58		51	50	
	Relatives’ house	2	5		2	4		3	3	
	Organisation house	2	1		2	2		2	2	
Economic status	Low	31	33	0⋅42	31	35	0⋅53	32	34	0⋅26
	Medium	49	88		61	82		66	77	
	Good	40	41		41	32		41	32	
Smoking	Yes	7	9	0⋅78	9	7	0⋅54	7	9	0⋅47
	No	107	159		129	137		141	125	

χ^2^ test for recognising confounders into categories. cat1: healthy mental; cat2: unhealthy mental.

Table 3 illustrates the mean ± sd of characteristics of variables in four categories, showing that there are significant body composition differences, including weight, BMI, waist circumference^(27)^, fat-free mass^(29)^, VFA, TG (*P* < 0⋅0001) and CHOL (*P* < 0⋅05), and diastolic blood pressure (*P* < 0⋅02).

**Table 3.** Characteristics of variables in four categories

Variable	Q1		Q2		Q3		Q4		*P* value

	Mean (77)	SD	Mean (31)	SD	Mean (82)	SD	Mean	SD	
Age (years)	35⋅48	8.02	33⋅70	8.15	37⋅60	8.92	37⋅04	7.98	0⋅11
Weight (Kg)	71⋅85	5.32	74⋅37	8.19	89⋅22	11.31	87⋅05	11.35	<0⋅001
BMI (kg/m^2^)	27⋅51	1.47	27⋅79	1.44	34⋅28	3.85	33⋅76	3.64	<0⋅001

Body composition analysis									
Body fat mass	27⋅38	3.38	28⋅58	4.40	40⋅17		39⋅29	7.19	<0⋅001
FFM (%)	44⋅39	4.13	45⋅46	5.65	49⋅17	5.59	48⋅43	5.44	<0⋅001
WHR	0⋅90	0.04	0⋅91	0.04	2⋅05	9.99	0⋅96	0.04	0⋅59
WC	91⋅46	5.05	93⋅34	6.72	105⋅74	8.29	105⋅66	8.63	<0⋅001
VFA (cm^2^)	133⋅71	22.37	141⋅54	28.48	206⋅72	182.70	189⋅08	29.73	<0⋅001

Blood parameters									
Cholesterol (g/dl)	175⋅98	29.07	184⋅34	33.00	192⋅87	41.93	188⋅48	37.46	0⋅05
TG (g/dl)	99⋅64	46.63	103⋅10	42.22	145⋅46	92.03	124⋅60	74.41	<0⋅001
LDL (mg/dl)	91⋅60	19.98	93⋅06	26.43	95⋅68	25.03	95⋅11	26.00	0⋅77
HDL (mg/dl)	47⋅42	10.11	47⋅20	11.16	44⋅69	11.23	47⋅00	12.82	0⋅51
SBP (mm Hg)	110⋅20	11.74	108⋅62	13.85	113⋅42	15.47	114⋅92	13.50	0⋅12
DBP (mm Hg)	77⋅23	8.59	74⋅40	8.46	78⋅60	9.97	81⋅07	11.41	0⋅02

BMI; body fat mass, VFA; visceral fat area; FFM, fat-free mass; WHR, waist hip ratio; WC, waist circumference; TG, triacylglycerol; HDL, high-density lipoprotein; LDL, low-density lipoprotein; SBP, systolic blood pressure; DBP, diastolic blood pressure. Q1, overweight without D deficiency; Q2, overweight with D deficiency; Q3, people with obesity without D deficiency; Q4, people with obesity D deficiency.

The ANOVA test was performed for the potential effect of confounders in each quartile.

Table 4 shows the evaluation relationship between mental health and DBM, where there were no observed significant associations between anxiety and depression after adjusting potential confounders including age, energy and marital status in model 1 (0⋅26, 0⋅74) *v.* the crude model (0⋅35, 0⋅75), respectively. However, there was a significant relationship between stress and DBM after adjusting age, energy and marital status in model 1 (0⋅04) *v.* the crude model (0⋅09).

**Table 4.** Comparison association between DBM and mental health before and after adjusting potential confounders

Variables	Q1	Q2	Q3	Q4	*P*-trend
Anxiety					
Model 0	1	1⋅12 (0⋅48–2⋅58)	1⋅97 (1⋅00–3⋅86)	1⋅01 (0⋅46–2⋅21)	0⋅35
Model 1	1	1⋅32 (0⋅55–3⋅19)	1⋅87 (0⋅93–3⋅76)	1⋅21 (0⋅54–2⋅73)	0⋅26
Stress					
Model 0	1	1⋅45 (0⋅63–3⋅34)	1⋅54 (0⋅80–2⋅95)	1⋅84 (0⋅83–4⋅05)	0⋅09
Model 1	1	1⋅38 (0⋅58–3⋅29)	1⋅53 (0⋅78–3⋅02)	2⋅29 (1⋅00–5⋅22)	< 0⋅04
Depression					
Model 0	1	0⋅79 (0⋅34–1⋅84)	1⋅05 (0⋅55–2⋅01)	1⋅08 (0⋅49–2⋅35)	0⋅75
Model 1	1	0⋅72 (0⋅30–1⋅74)	0⋅98 (0⋅50–1⋅93)	1⋅13 (0⋅50–2⋅54)	0⋅74

Model 0: crude model; Model 1: adjusted for age, energy, marriage status.

## Discussion

This novel investigation sought to identify the role of vitamin D as a moderator in the association between DBM and mental health and, to our knowledge, was the first conducted in Iranian women. In the present study, we considered the presence of overweight and obesity with the deficiency of vitamin D as a DBM. Many previous studies have demonstrated a significant association between vitamin D and depression. Moreover, vitamin D levels have been shown to be impeded due to air pollution in industrial cities, low intake in some countries, use of cosmetics, sunlight cream, some clothing because of religion and age because of decreasing synthesis by skin and absorption by the intestine. Iranian women are reported to have an increased susceptibility to vitamin D deficiency, either by type of clothing or low consumption of dairy. In the present study, we observed a significant relationship between DBM and stress; in addition, we observed significant differences between four categories in BMI, weight and body composition items including waist circumference^(27)^, fat-free mass^(29)^, VFA and lipid profiles, such as TG and CHOL, and diastolic blood pressure.

Vitamin D is an important fat-soluble vitamin that is immune-modulating and can inhibit many communicable diseases including, heart disease, multiple sclerosis, rheumatoid arthritis and type 1 diabetes. Indeed, the deficiency of vitamin D is associated with many disorders, including skeletal abnormalities, delayed growth in children, osteoporosis and osteopenia, reduction of calcium and phosphorus absorption in the intestine. The main sources of vitamin D are exposure of skin to sunlight and consumption of foods containing vitamin D, such as dairy. Vitamin D deficiency is common in Iranian people; indeed, based on a meta-analysis that was performed in Iranian people, 45⋅64 % of men and 61⋅90 % of women showed vitamin D deficiency. Moreover, the geographical location, type of clothing, poor diet, expensive dairy costs and skin colours are posited to modulate this deficiency^(39–43)^.

25(OH)D levels, either by diet or synthesised by skin, are highly dependent on vitamin D binding protein (DBP), also a high level of 25(OH)D, as well as 1⋅25(OH)D, appear on DBP (more than 80 %), whereas DBP is responsible for 25(OH)D plasma concentrations. Some studies have reported a positive association between BMI and DBP plasma concentration, and many studies have reported that the 25(OH)D concentrations in overweight and obese people may be reduced significantly. Because of this evident correlation between vitamin D status and DBP in obese individuals, we considered the level of plasma vitamin D status between 20 and 30 ng/l and less than 20 ng/ml to represent insufficiency and deficiency, respectively^(34,35,44,45)^.

Currently, obesity and overweight represent significant health issues associated with inflammatory status. Obesity is implicated in a mutation in the leptin/melanocortin pathway in the central nervous system, which plays a role in regulating body energy haemostasis. Moreover, obesity related to many brain disorders and psychopathology conditions, including eating and mood disorders^(46–48)^, plays an important role in depression conditions^(38,49–51)^, where high BMI has been associated with mood disorders (OR 1⋅23) and major depression (OR 1⋅27). In addition, evidence suggests a significant relationship between high BMI and anxiety disorders, such as post-traumatic stress disorders (OR 2⋅64)^(52)^. Anglin *et al.* performed a systematic review and meta-analysis on one case control, three cohorts, and ten cross-sectional studies and reported a significant relationship between vitamin D deficiency and depression^(10)^.

Concordant with our findings, Mousa *et al.* carried out a study on sixty-three overweight participants (thirty-nine males and twenty-four females), with a mean age = 31⋅3 ± 8⋅5, along with vitamin D deficiency [25(OH)D < 20 ng/l] and BMI 25 kg/m^2^, without clinical depression, consumed a bolus oral dosage of 100 000 IU, followed by 4000 IU daily cholecalciferol for 16 weeks. Subsequently, the authors observed an increasing concentration of plasma vitamin D, and reported that vitamin D deficiency was not associated with depression, has therefore not warranted for reducing depression symptoms^(53)^.

In contrast with our finding, Schaad *et al.*, in 18–67 years old males and females, found a significant relationship between vitamin D deficiency and depression. However, the number of males (*n* 9799) was notably more than the number of female counterparts (*n* 1121), which likely biased the findings^(54)^. Similarly, Umhau reported a significant correlation between suicide and vitamin D deficiency among active-duty military personnel, where the number of males (*n* 467) was significantly greater than the number of females (*n* 19)^(26)^. Nevertheless, tentative conclusions from the literature suggest that the correlation between depression and vitamin D deficiency may be greater in males *v.* females.

Although we present a novel addition to the literature, where we assessed DBM and mental health in Iranian women, there were several limitations that should be noted. First, we did not assess normal-weight participants with vitamin D deficiency, this precluded a comparison between normal weight *v.* overweight/obesity and represents a sensible avenue for further research. Moreover, although we focused on women, who appear to be relatively underrepresented in comparable research, future studies should be designed considering an equitable split in sex.

## Conclusion

In this cohort study, we demonstrated that stress and DBM were significantly associated; however, the directionality of the relationship remains unclear. Vitamin D insufficiency is associated with mental health and obesity in opposing directions, and whether vitamin D supplementation can improve mental health impairments requires further evaluation.
